# Evolutionary conservation of nested MIR159 structural microRNA genes and their promoter characterization in *Arabidopsis thaliana*

**DOI:** 10.3389/fpls.2022.948751

**Published:** 2022-07-26

**Authors:** Muhammad Imran, Tengfei Liu, Zheng Wang, Min Wang, Shulin Liu, Xinyan Gao, Anning Wang, Songfeng Liu, Zhixi Tian, Min Zhang

**Affiliations:** ^1^State Key Laboratory of Plant Cell and Chromosome Engineering, Institute of Genetics and Developmental Biology, Innovative Academy for Seed Design, Chinese Academy of Sciences, Beijing, China; ^2^Beijing Vegetable Research Center (BVRC), Beijing Academy of Agriculture and Forestry Sciences (BAAFS), Beijing, China; ^3^University of Chinese Academy of Sciences, Beijing, China

**Keywords:** *Arabidopsis*, *MIR159*/miR159, nested miRNA structure, evolutionary analysis, promoter analysis, expression profile

## Abstract

MicroRNAs (miRNAs) are endogenous small RNAs, that are vital for gene expression regulation in eukaryotes. Whenever a pri-miRNA precursor includes another miRNA precursor, and both of these precursors may generate independent, non-overlapping mature miRNAs, we named them nested miRNAs. However, the extent of nested miR159 structural evolutionary conservation and its promoter characterization remains unknown. In this study, the sequence alignment and phylogenetic analysis reveal that the MIR159 family is ancient, and its nested miR159 structures are evolutionary conserved in different plant species. The overexpression of ath-MIR159a, including the 1.2 kb downstream region, has no effect on rescuing the *mir159ab* phenotype. The promoter truncation results revealed that the 1.0 kb promoter of ath-MIR159a is sufficient for rescuing the *mir159ab* phenotype. The *cis*-regulatory elements in the ath-miR159a promoters indicated functions related to different phytohormones, abiotic stresses, and transcriptional activation. While the MybSt1 motif-containing region is not responsible for activating the regulation of the miR159a promoter. The qRT-PCR results showed that overexpression of ath-MIR159a led to high expression levels of miR159a.1–5 and miR159a.1–3 and complemented the growth defect of *mir159ab via* downregulation of *MYB33* and *MYB65*. Furthermore, continuously higher expression of the miR159a.2 duplex in transgenic lines with the curly leaf phenotype indicates that miR159a.2 is functional in *Arabidopsis* and suggests that it is possible for a miRNA precursor to encode several regulatory small RNAs in plants. Taken together, our study demonstrates that the nested miR159 structure is evolutionary conserved and miRNA-mediated gene regulation is more complex than previously thought.

## Introduction

MicroRNAs (miRNAs) are a type of short RNAs with a length of roughly 21 nucleotides (nt) that have been identified as key regulators of gene expression (Voinnet, 2009), emerged and specialized independently in both animals and plants, due to differences in their biogenesis (Axtell et al., 2011). During the biogenesis, miRNAs are made up of lengthy precursors with an incomplete foldback structure, and small RNA is inserted in one of their arms. Further, these precursors include spatial cues that are detected during the biogenesis of the small RNAs (Bologna and Voinnet, 2014; Ha and Kim, 2014). Plant miRNA precursors can be very different than their animal counterparts, and DICER-LIKE1 (DCL1) is part of complex that processes plant miRNA precursors in the nucleus (Axtell et al., 2011; Rogers and Chen, 2013; Bologna and Voinnet, 2014). With the help of HYPONASTIC LEAVES 1 (HYL1) and SERRATE, each miRNA precursor is processed by DCL1 enzyme through two consecutive cleavage reactions to form a single small duplexed-RNA comprising the miRNA and its partly complementary strand (Bartel, 2004; Meyers et al., 2008). The small RNAs, including miRNAs and siRNAs, are further modified by HEN1 at 3′ end through its RNA methyl transferase activity and exported into the cytoplasm (Yu et al., 2005). Then, miRNAs bind to the complementary sites on target mRNAs after being loaded into the RNA-Induced Silencing Complex (RISC), causing translational repression and/or cleavage of target mRNAs to occur (Bartel, 2004).

In recent years, it has been noted that plant miRNAs conservation in distant species is most clearly observed in the miRNA/miRNA* region (Reinhart et al., 2002). A structural property of miRNAs is that their precursors form fold-back hairpin structures. A miRNA precursor is generally expected to produce one miRNA-miRNA* duplex (Bartel, 2004; Kim, 2005; Winter et al., 2009). Though earlier studies discovered additional small RNAs in addition to the miRNAs and the miRNA*s, these additional small RNAs were generally assumed to be by-products of Dicer activity and have never been carefully examined (Kurihara and Watanabe, 2004; Rajagopalan et al., 2006; Ruby et al., 2007; Lacombe et al., 2008). However, the ancient miR319 precursor contains a second conserved region on the precursor stem above the miRNA/miRNA* (Addo Quaye et al., 2009; Bologna et al., 2009; Li et al., 2011; Sobkowiak et al., 2012), demonstrating the presence of extra conserved sequences in at least some MIRNAs. Furthermore, Zhang et al. found 19 miRNA precursors in *Arabidopsis*, each of which can produce many different miRNA-like RNAs in addition to miRNAs and miRNA*s (Zhang et al., 2010). In a deep-sequencing-based study of small RNAs (Liu et al., 2016), we identified that many miRNA precursors were completely included in another miRNA precursor, and we called them nested miRNA structures. In our study, *MIR159* precursors also had a nested structure. Previous studies revealed that the MIR159 precursor is unusually lengthy, and other small RNAs derived from it have been discovered in *Arabidopsis* by large-scale sequencing (Fahlgren et al., 2007) and genome-scale analyses (Zhang et al., 2010; Li et al., 2011). However, the nested structure conservation in kingdom planta is still unknown.

To investigate the conservation of the nested MIR159 family in terrestrial plants, we first collected and examined a large number of MIR159 stem-loops from various plant species. Then, using RNA models for paired and unpaired nucleotides, we reconstructed the phylogenetic tree of MIR159 from a structural alignment, showing the evolutionary history of this miRNA gene family. Secondly, *MIR159* is tightly involved in plant development and highly conserved in many plant species, including angiosperms, mosses, and lycopods (Rhoades et al., 2002; Li et al., 2014). In *Arabidopsis*, miR159 family targets MYB which has been extensively examined as a framework for miRNA-mediated gene silencing in plants (Vella et al., 2004; Allen et al., 2010). However, a genetic study showed that only *MYB33* and *MYB65* were functionally targeted by miR159, and *mir159ab* developmental abnormalities were reversed in a *myb33mir159ab* quadruple mutant (Allen et al., 2007). Therefore, we used *mir159ab* as a model to investigate the regulatory function of up- and downstream region of miR159a *via* overexpressing the miR159a with 1.2 kb downstream region and promoter truncations to rescue the *mir159ab* double mutant phenotype.

## Materials and methods

### Plant materials and growth conditions

Seeds of the *Arabidopsis mir159ab* mutant were provided by Professor Anthony A. Millar (Research School of Biology, Australian National University, Australia). Wild-type Columbia-0 (Col-0) and *mir159ab* mutant plants were grown in the greenhouse of the Institute of Genetics and Developmental Biology, Chinese Academy of Sciences. Plants were grown in growth rooms at 22°C with 16 h of light and 8 h of darkness. Size parameters were measured with ImageJ software.^1^

### Search of nested *MIR159*/miR159 gene

Nested *MIR159*/miR159 genes in soybean were identified by following the previous method (Liu et al., 2016). Then, precursor sequences of MIR159 were gathered from the miRbase Version 11.0 (Griffiths Jones et al., 2006). The secondary structures of RNA were predicted using the RNAfold program (Hofacker et al., 1994). This three-letter code shown in all lower case is used for sequences from miRbase (Griffiths Jones et al., 2006). The taxonomic tree was created with the assistance of the NCBI taxonomy and drawn by NJplot (Perriere and Gouy, 1996).

### Sequence alignment and phylogenetic tree analysis

Alignment of all MIR159 stem-loop sequences was performed using the T-Coffee software version 6.06 (Notredame et al., 2000). Each sequence in the alignment was used to generate secondary structures, which were created using RNAfold (Hofacker et al., 1994). The slogo program was used to create the structure logos (Gorodkin et al., 1997). The phylogenetic tree was constructed with MEGA6 software using NJ method (Tamura et al., 2013).

### Construction of MIR159a-3′UTR overexpression vector

To obtain 2 × 35S::ath-MIR159a-3′UTR transgenic plants, 1.2 kb genomic sequence immediately downstream of the *ath-MIR159a* stem-loop was amplified by PCR from *Arabidopsis* DNA using primer pairs and cloned into the vector pMDC32 (Invitrogen). All constructs in this report, including the gateway entry and destination vectors, were created using standard cloning techniques and then transformed into the *mir159ab* background. Transgenic plants were selected with 40 mg/l hygromycin.

### Generation of truncated promoters and MybSt1 element mutated constructs

To understand the regulatory mechanism of *MIR159a*, truncated promoter constructs with different lengths (3.0, 2.5, 2.1, 1.5, 1.0, 0.6, and 0.2 kb) of *ath-MIR159a* upstream sequences and 1.2 kb downstream of the *ath-MIR159a* stem-loop were amplified from *Arabidopsis* and cloned into the pMDC99 vector (Invitrogen). The cis-regulatory element analysis was performed using PALACE database.^2^ The deletion constructs Δ858–862, Δ946–950, and Δ858/Δ946 were created according to the manual of the Fast Mutagenesis Kit (TransGen Biotech, Beijing, China). Primers specific for each construct were designed by Primer Premier 5.0 (PREMIER Biosft, Palo Alto, CA, United States) and are listed in Supplementary Table S1.

### Transformation and phenotypes measurement of *Arabidopsis*

In this study, all the vectors were electroporated and transferred into *Agrobacterium tumefaciens* strain GV3101 (Hellens et al., 2000). *Agrobacterium tumefaciens* cells containing overexpressing and promoter truncation variants were harvested by centrifugation at 5,000×g for 8 min and resuspended in 5% sucrose solution to a final OD of 0.5. By using the floral dip method (Steven and Andrew, 1998), the shoot apex of *Arabidopsis mir159ab* double mutant plants was dipped into a bacterial suspension supplemented with 0.05% Silwet (Silwet L-77, Sigma). Seeds were germinated on agar plates containing Murashige and Skoog basal medium as well as antibiotics in order to select transformants from the population. Transformants were detected and transplanted into the soil after 7 to 10 days of growth. The photographs of all plants were taken with the help of DSLR EOS 70D (Canon) using scale as reference. Then, the size of a rosette, lamina length, lamina width, and leaf area of plants were measured with ImageJ (version 1.8.0) software.

### RNA extraction and qRT-PCR

With the help of the TRIzol reagent (Invitrogen, United States), total RNA was isolated from plants at different stages of growth. The purity of the RNA was then checked by agarose gel electrophoresis and quantified with a Nanodrop 2000 spectrophotometer (Thermo Fisher Scientific, Waltham, MA, United States). For quantitative detection of the genes, cDNA was first synthesized using M-MLV (Promega, United States) and detected with TransStart Tip Green qPCR SuperMix (TransGen Biotech, China). For comparison of sibling mature miRNA, total RNA was purified with the miRcute miRNA purification kit (Tiangen Biotech, China) and reverse transcribed to cDNA with a miRcute Plus miRNA first-strand cDNA synthesis kit (Tiangen). A minute Plus miRNA qPCR kit (SYBR Green) was used for qRT-PCR by following the manufacturer’s protocol in a total volume of 20 μl. All qRT-PCRs (for both reference and genes of interest) were carried out on a Rotor-Gene Q Real-time PCR machine in triplicate under the following cycling conditions: 1 cycle of 95°C/5 min, 45 cycles of 95°C/15 s and 60°C/15 s, and fluorescence was analyzed at 72°C/20 s. A 55°C to 99°C melting cycle was then carried out. *CYCLOPHILIN* (*At2g29960*) was used to normalize mRNA levels, and all sibling mature miR159 levels were normalized to U-6. The value for each gene represents the average of triplicate assays. The 2^−ΔCt^ method for relative quantification of gene expression was used to determine the level of miRNA expression.

### Statistical analysis

The one-way ANOVA with the SPSS 11.5 package for Windows (SPSS, Inc., Chicago, IL, United States) was used for statistical analysis in this work. The Student’s *t*-test was used to examine the differences between the two groups of data. Results with a corresponding probability value of *p* < 0.05 and *p* < 0.01 were considered to be statistically significant and very significant, respectively.

## Results

### The nested miR159 structure is highly conserved in the plant kingdom

**Figure 1:**
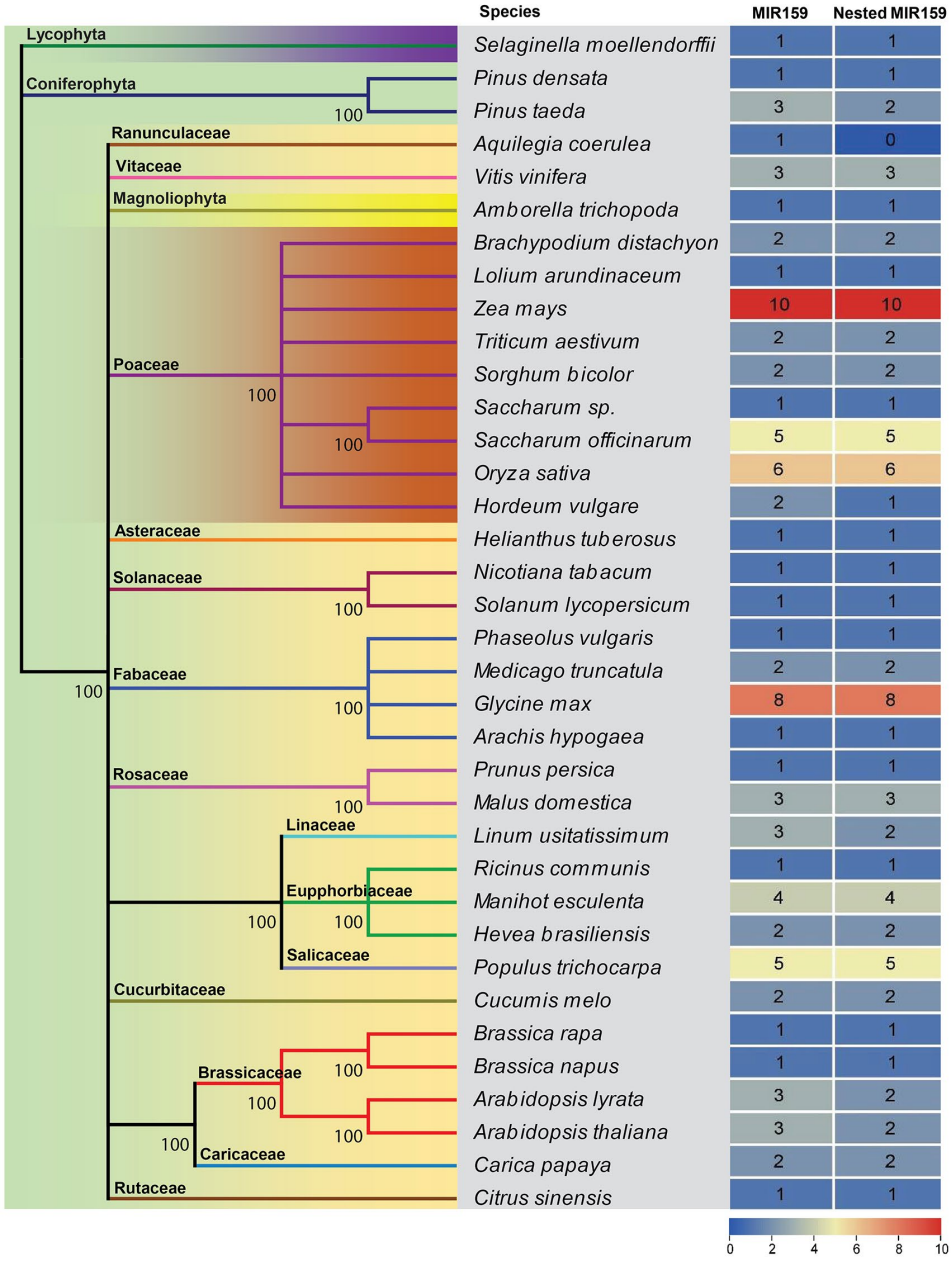
Evolutionary conservation of *MIR159* and the nested *MIR159* structure. A phylogenetic tree of *MIR159* and nested *MIR159* structure was constructed from 36 green plant species represented in miRBase release 21 databases.

When a miRNA precursor includes another miRNA precursor and both of these precursors may generate independent non-overlapping mature miRNAs, we designated them nested miRNAs. It has been recently shown that several miRNAs are conserved over large evolutionary distances from embryophytes to core rosids, and few miRNAs are specific to species or lineages (Axtell and Bartel, 2005; Cuperus et al., 2011; Montes et al., 2014). However, during evolution, mutations acquired in miRNA stem-loops can provide valuable information for inferring the phylogeny of miRNA families with ancient origins (Axtell and Bowman, 2008; Cuperus et al., 2011). To determine whether the Brassicaceae lineage has a similar arrangement conserved in other species, we analyzed pri-miR159a sequences annotated in miRBase.^3^ After the analysis, 82 out of 88 MIR159 genes from 36 land plant species had nested structures. The *MIR159* genes are conserved from mosses to flowering plants, and their stem-loop precursors usually have elongated stem structures. The miR159 family is a deeply conserved miRNA family and universally expressed among diverse land plants (Figure 1). Except *Aquilegia coerulea*, all the plants expressing the miR159 family had nested structures (Figure 1), indicating that nested miR159 structures were ancient.

**Figure 2:**
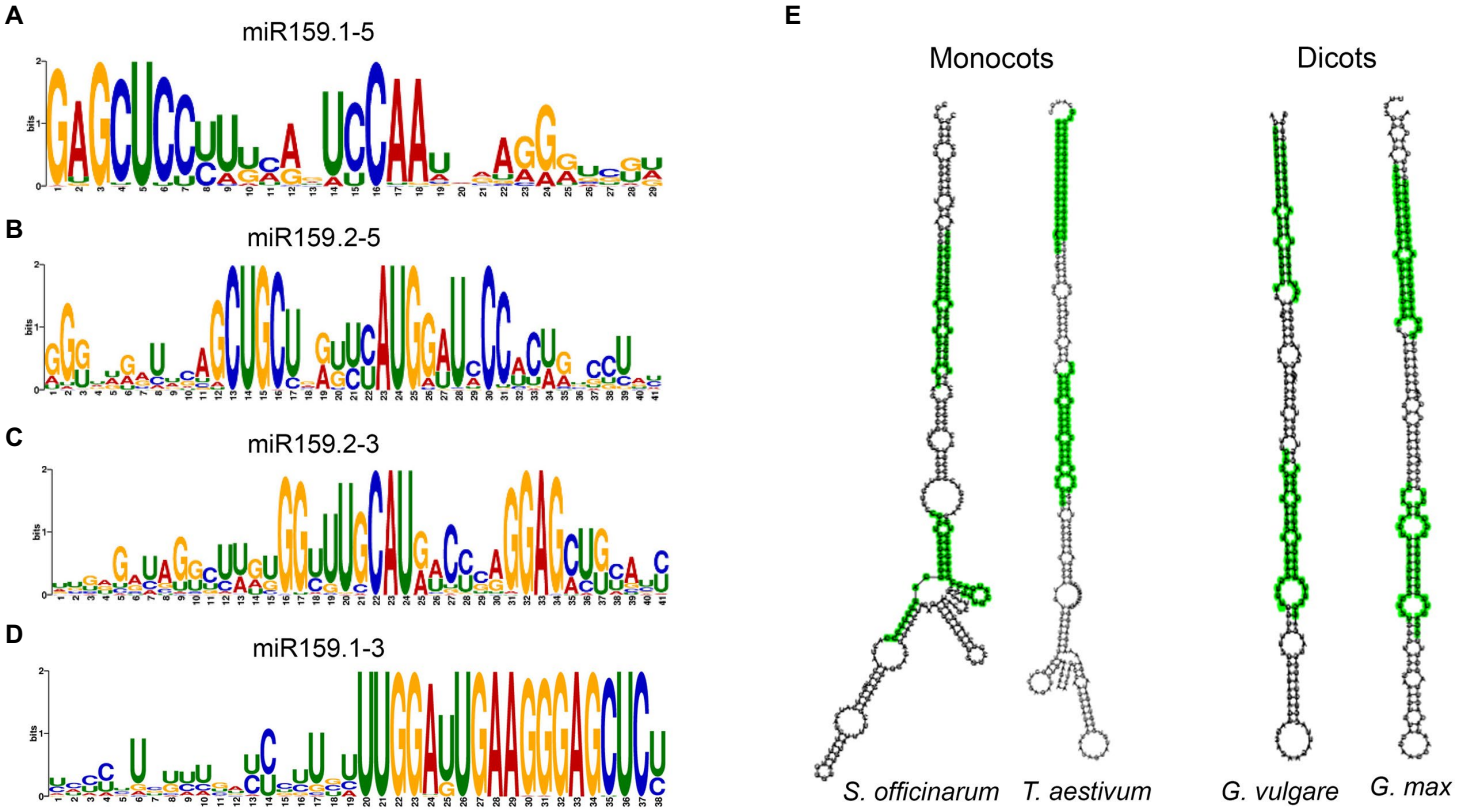
Core sequences of sibling mature miRNA Sequences. **(A–D)** Conservation profile of foldback sequences of all the *MIR159* genes with sibling mature miRNA sequences shown by MEME motif. **(E)** Monocots and dicots miR159 nested structures. Green: sibling miRNAs got by blastn search or MEME motif discovery.

Using the MUSCLE program to align the sequences obtained from various plant species, we aligned the MIR159 stem-loops considering both sequences and observed that 85% of sibling mature miR159a.1 and miR159a.2 duplex regions are highly conserved among nested miR159a members from different organisms (Figures 2A–D; Supplementary Figure S1), demonstrating that sibling mature miR159 sequences are highly conserved among different plants. In addition to the conserved sibling mature miRNAs, 9% of mature sibling miRNAs were specific (Supplementary Table S2). The aligned predicted miR159 family member secondary structure determined by RNAfold software revealed that 83% of nested miR159a had conserved secondary structure patterns similar to those of ath-miR159. Furthermore, the difference of monocots and dicots nested structures with elongated stem-loop and branches is presented in Figure 2E. However, few locations in the loop and other double-stranded regions of the precursor were less conserved, implying less selective pressure. The overall sequence of miR159 has been maintained during plant evolution, with only a few minor modifications that make for simple alignment of small RNA sequences (Supplementary Figure S1).

**Figure 3:**
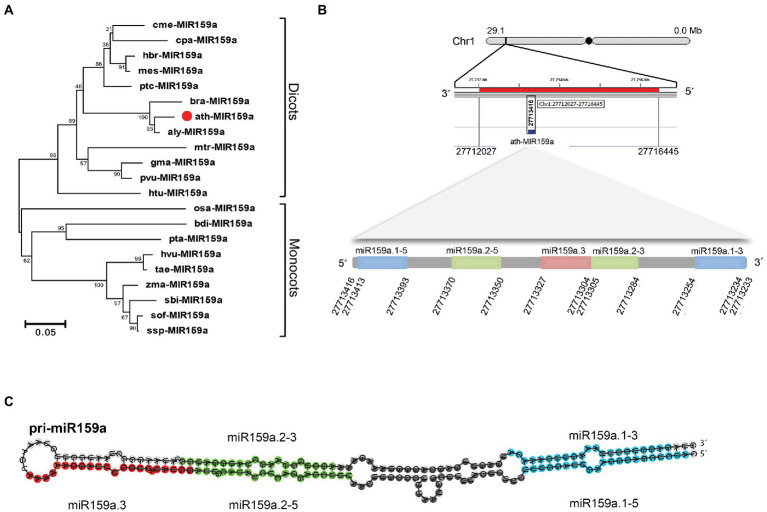
Phylogenetic tree and genomic loci of pri-miR159a. **(A)** Phylogenetic tree of miR159a from dicotyledonous and monocotyledonous plants. **(B)** Genomic position of ath-miR159a. **(C)** Nested miR159a secondary structure of Arabidopsis; Blue, green, and red colour indicate the position of duplex miR159a.1 (containing sibling’s miR159a.1–5 and miR159a. 1–3, that are located on 5 and 3 prime arm of miR159a secondary structure, respectively), miR159a.2 (Containing sibling’smiR159a.2–5 and miR159a. 2–3, that are also located on 5 and 3 prime arm of miR159a secondary structure, respectively) and miR159a.3, respectively. This figure is obtained from our previous published paper (Imran et al., 2022).

As we observed, not all MIR159 precursors have conserved elongated stems and can be well aligned in loop-proximal regions because different miRNA family members have different secondary structures and sequence lengths, as shown in Figure S1. Further investigation of the miR159a precursor sequence obtained from various species using the T-Coffee program (Notredame et al., 2000) showed that the flanking sequence was variable, but the nested mature sequences of miR159a.1 and miR159a.2 were similar, confirming the structural conservation of *MIR159* precursors (Supplementary Figure S2). Phylogenetic analysis of miR159a precursors revealed that ath-miR159a, aly-miR159a, and bra-miR159a were clustered together, while monocots and dicots were divided into two groups due to sequence conservation (Figure 3A). Additionally, RNAfold software was used to predict the stem-loop secondary structure of miR159a members to confirm the difference in the clustering group. The secondary structure analysis results revealed that miR159a possesses two major types of structures, one each for monocots and dicots. Monocot species possess a single elongated stem-loop, while dicots possess different branches near the loop region but have conserved nested miR159a structures (Supplementary Figure S3). Overall, these findings indicate that pri-miR159a encodes a second microRNA that is conserved across plant species in terms of sequence and location, as well as the stem-loop structure, and indicates the origin of MIR159 from a long stem-loop.

We further investigated nested structures in *Arabidopsis* and identified six groups of nested miRNAs in *Arabidopsis thaliana* containing 18 mature miRNAs (Supplementary Table S3) and classified them into three groups (Zhang et al., 2010). These nested miRNA structure groups are scattered in the genome with forward and reverse orders at different locations, and the majority of them possess 20 nucleotides except miR4471–3p and miR829a.1, which have 21 and 23 nucleotides, respectively (Supplementary Table S3). Additionally, the pre-miRNA coordinate for MIR159a is 808 bp long and ranges from 27,713,700 to 27,712,893 in reverse order according to TAIR10 based on chromosome one in *A. thaliana* (Figure 3B). While the nested miR159a structures are located between 27,713,416 and 27,713,234 bp, we further designated them miR159a.1–5, miR159a.2–5, miR159a.3, miR159a.2–3, and miR159a.2–5 based on their foldback secondary structure, which starts from the 5′-end of the precursor (Figure 3B). Each mature sequence precursor of the nested miR159a structure has 21 nucleotides, except miR159a.3, which possesses 20 nucleotides. While, the nested miR159a structure is presented in Figure 3C.

### Role of ath-miR159a-3′UTR on rescuing the *mir159ab* phenotype

**Figure 4:**
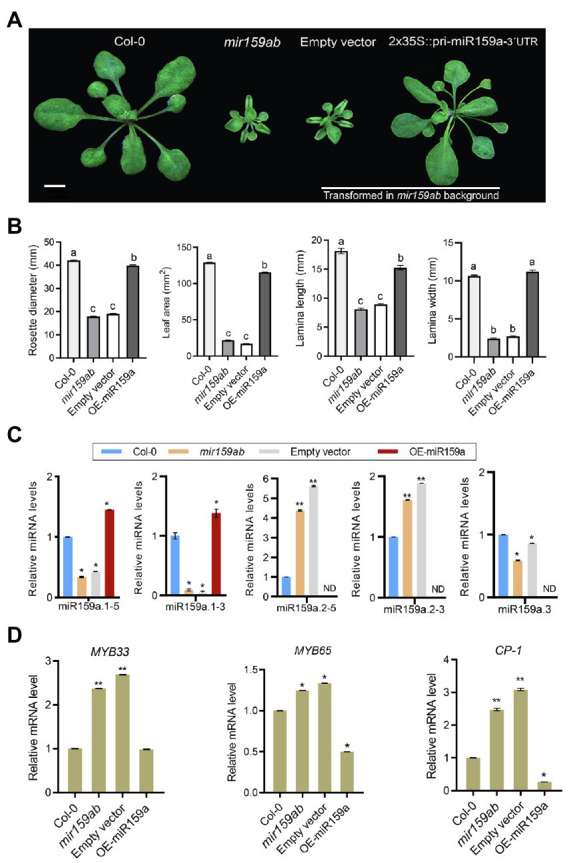
Pri-miR159a-3′UTR overexpression. **(A)** Phenotypes of 22-day-old wild-type (Col-0), *mir159ab*, empty vector, and OE-miR159a-UTR plants. Scale bar: 1 cm. The empty vector and OE-miR159a-3UTR constructs were transformed into the *mir159ab* background. **(B)** The rosette size and leaf lamina length, lamina width, and leaf area from Col-0, empty vector, and overexpressed plants were evaluated. For measurement, the fourth true leaf of 28-day-old plants was collected. Data were presented as the mean ± SE (*n* > 10) and analyzed with one-way ANOVA and multiple comparisons (*p* < 0.05, marked with different characters). **(C)** miRNA and **(D)** mRNA relative expression levels were normalized to U-6 and *CYCLOPHILIN*, and the relative expression in the wild type was set as 1. Measurements are the average of three technical replicates. Error bars represent the SEM. Asterisks indicate statistically significant differences at *p* < 0.05 (^*^) and *p* < 0.01 (^**^) by Student’s *t*-test.

3′ regulatory regions play significant roles in gene transcription termination processes such as cleavage and polyadenylation (Rosenthal et al., 2018). Indeed, as evidenced by the usage of various 3′ regulatory regions in expression cassettes, 3′ regulatory regions have a major impact on gene expression levels (Hirai et al., 2011; Diamos and Mason, 2018; Rosenthal et al., 2018). Further, a C-to-T substitution in the second motif CNS2 of 3′UTR increased the URL1 mRNA stability and affected the leaf phenotype in rice (Fang et al., 2021). Besides, recent studies have revealed roles for miRNA sequences beyond the seed region in specifying target recognition and regulation (Chipman and Pasquinelli, 2019), suggesting more complex mechanisms of protein expression control. However, in contrast to miRNA 5′ (target complementarity) region, the 3′ region significance in miRNA-target identification is less clear. To understand the function of the nested miR159a 3′UTR in *Arabidopsis* and to determine whether overexpression of *ath-MIR159a,* including the 1.2 kb region, could interfere with the curly leaf growth process, we created *ath-MIR159a* overexpression lines driven by the CaMV 35S promoter (Figure 4A) and transformed them into the double mutant *mir159ab*. All 26 independent T1 transgenic plants showed a rescued *mir159ab* phenotype (Figure 4A). The overexpressed plants exhibited a longer lamina width, rosette diameter, leaf area, and laminae length and rounded leaf blade than the empty vector and were more similar to the wild type (Figure 4B). To investigate whether pri-miR159a was properly processed into mature miRNA, we checked the expression levels of sibling mature miRNAs through qRT-PCR analysis and found that miR159a.1–5 and miR159a.1–3 levels in the overexpressed line were significantly higher (Figure 4C). Thus, the miR159a.1 duplex was successfully expressed in the transgenic plants. However, miR159a.2–5 and miR159a.2–3 showed sixfold and twofold higher expression levels, respectively, in the empty vector than in the wild type and were not expressed in overexpression plants (Figure 4C), indicating that miR159a.1 may negatively regulate the expression of miR159a.2. Since *MYB33* and *MYB65* are the only two targets of miR159a, to test whether they are under miR159 control, we quantified the expression of *MYB33* and *MYB65* genes in 3-week-old rosettes of wild-type, *mir159ab*, and OE-miR159a plants. The expression of *MYB33* and *MYB65* was dramatically suppressed in the OE-miR159a plants compared to the empty vector (*mir159ab*); moreover, the expression level of *MYB33* was similar to that in the wild type, but *MYB65* was deregulated compared to that in the wild type (Figure 4D). Consistent with the *MYB65* level, the expression of downstream *GAMYB-like CYSTEINE PROTEINASE1* (*CP1*) gene in OE-miR159a plants was also reduced (Figure 4D). These results showed that overexpression of *ath-MIR159a* resulted in high expression levels of miR159a.1–5 and miR159a.1–3 and complemented the growth defect of *mir159ab via* downregulation of *MYB33* and *MYB65.* However, overexpression of *ath-MIR159a* did not fully rescue the *mir159ab* phenotype, indicating that the precursor is being expressed in regions where is not normally there or miR159a.2 or miR159a.3 may regulate some potential unknown targets.

### The 1.0 kb promoter of ath-miR159a is sufficient for rescuing the *mir159ab* phenotype

Promoters are crucial for activating gene transcription and controlling transgene expression. Therefore, for the efficient implementation of transgenic breeding and gene function studies, a thorough understanding of promoter transcriptional activities and expression levels is required (Hernandez Garcia et al., 2009). Therefore, to understand how ath-miR159a is activated to express and determine whether these two *MIRNAs* are driven by the same promoter or two different promoters, the 3.0 kb promoter sequence of *ath-MIR159a* (*AT1G73687.1*) upstream of MIR159a precursor sequence and the 1.2 kb sequence downstream of MIR159a precursor sequence were isolated from *A. thaliana* genomic DNA based on the public sequence from TAIR.^4^ As deregulated MYB33/65 activity by miR159 is tightly correlated with the extent of upward leaf curl (Allen et al., 2007), this trait was used to visually assess the strength of the complementation of the *mir159ab* phenotype by each promoter construct. Furthermore, rosette diameter, leaf area, lamina length, and lamina width in transgenic plants were examined. By using this strategy, we analyzed promoter length of miR159a required for regulation in *A. thaliana*.

**Figure 5:**
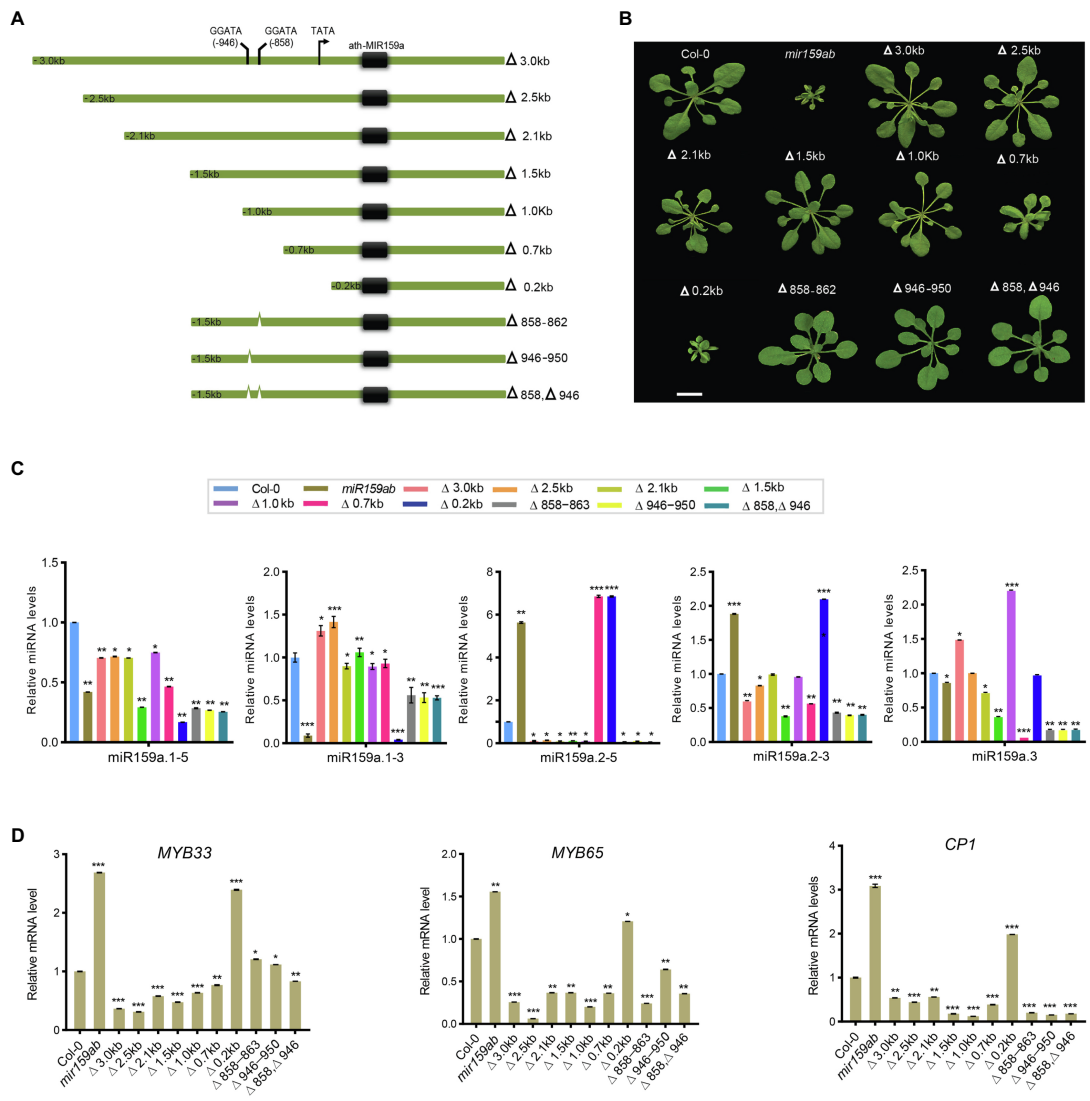
Pri-miR159a promoter truncations, and *cis*-element mutation analysis. **(A)** Schematic showing miR159a deletions used to drive miR159a transgenic *Arabidopsis*. Numbers refer to the end of the deletions from the transcription start site TATA box (black arrow). *Cis*-acting regulatory elements predicted by PLACE are shown between −858 and −946 with two predicted MybSt1 motifs (GGATA). Green thick lines represent the miR159a promoter. Numbers indicate positions from the putative transcription start site. **(B)** Phenotypes of 22-day-old wild-type (Col-0), *mir159ab*, and truncated and deleted promoter transgenic plants. Scale bar: 1 cm. **(C)** miRNA and **(D)** mRNA relative expression levels were normalized to U-6 and *CYCLOPHILIN*. Measurements are the average of three technical replicates. Error bars represent the SEM. Asterisks indicate statistically significant differences at *p* < 0.05 (^*^), *p* < 0.01 (^**^) and *p* < 0.001 (^***^) by Student’s *t*-test.

To identify the potential promoter length necessary for driving MIRNA in the *miR159a* promoter, a full-length promoter (3.0 kb) and a series of its 5′-truncated fragments (2.5, 2.1, 1.5, 1.0, 0.6, and 0.2 kb) were constructed (Figure 5A; Supplementary Table S4), transformed into the *mir159ab* mutant, and examined in transgenic *Arabidopsis* to view their expression patterns (Figure 5B). At least four T1-independent transgenic lines were analyzed for each promoter. The results from T3 transgenic lines for each construct are shown in Figure 5B. The deletion promoter from 3.0 to 1.0 kb drove the *ath-MIR159a* constructs and all rescued the *mir159ab* phenotype, while the 700 bp promoter partially rescued the phenotype, and the less than 173 bp promoter did not rescue the *mir159ab* phenotype (Figure 5B). To characterize the phenotype in more detail, plants were grown in a growth chamber under long-day conditions at 22°C for 3 weeks, and their phenotype showed that compared to *mir159ab*, the transgenic plant with promoters from 3.0 to 1.0 kb were significantly rescued (Figure 5B).

Next, the expression levels of sibling mature miRNAs were measured through qRT-PCR analysis in transgenic plants with different lengths of truncated promoters (3.0, 2.5, 2.1, 1.5, 1.0, 0.7, and 0.2 kb) to test their role in miR159 regulation. We found that miR159a.1–3 was expressed at low levels in non-rescued plants (curly leaves) but showed higher expression in fully rescued plants than in the double mutant *mir159ab* (Figure 5C). Even though the expression level of miR159a.1–3 was higher than that of the wild type in 2.5 and 3.0 kb transgenic plants, the phenotype was fully rescued. In contrast, the expression levels of miR159a.2–5 and miR159a.2–3 were highest in the non-rescued curly phenotype but lowest in fully rescued plants. However, miR159a.3 was highly expressed in the 1 and 3.0 kb promoters only (Figure 5C). Next, mRNA quantities of *MYB33* were determined in transgenic plants with various promoters. The transcript levels of *MYB33* can be affected by at least two factors: first, the strength of transcription of the transgene and, second, the strength of miR159-mediated silencing through a translational repression mechanism (Li et al., 2014). Consistent with the transcript translational repression mechanism, all the truncated prompter plants with fully rescued phenotypes had low levels of *MYB33* and *MYB65* (Figure 5D). To further confirm that *mir159ab* plants were fully complemented by the miR159a promoter, downstream gene expression of *CP1* was also examined by qRT-PCR, as its mRNA is highly expressed in *mir159ab* due to the deregulation of *MYB33/MYB65*. The expression level of *CP1* in truncated promoter transgenic plants complemented by *mir159ab* was less than that of wild-type plants. The results showed that all the promoters greater than 1 kb resulted in a high expression level of miR159a.1–3 and complemented the growth defect of *mir159ab via* downregulation of *MYB33* and *MYB65* (Figure 5D).

### The MybSt1 motif-containing region is not responsible for activating the regulation of the ath-miR159a promoter

Furthermore, the progressive 5′ deletion analysis showed that the 0.2 kb promoter could not rescue the phenotype, while the 0.7 kb promoter could partly and the 1.0 kb promoter could fully rescue the *mir159ab* phenotype, suggesting that the 300 bp sequence between 0.7 and 1.0 kb is critical for the regulation of the *miR159a* promoter to fully rescue the *mir159ab* phenotype (Figure 5). Sequence analysis revealed the location of two MYB binding sites: one MybSt1 binding site is located between −858 and −862 (Figure 5A, Supplementary Table S4) and the other is between −946 and −950. We hypothesized that basal promoter activity may require these elements. To test this hypothesis, these MybSt1 binding sites regulate the expression level of miR159a to rescue the *mir159ab* phenotype, two mutated promoters with −Δ858 and −Δ946 were created by deleting the sequences between Δ-858 ~ 862 and Δ-946 ~ 950, respectively, and transferred into *mir159ab* (Figure 5A). The phenotype results showed that all the T1 transgenic plants had a rescued phenotype. Furthermore, we created two deletion constructs (Δ858–863 and Δ946–951) by excising the sequences corresponding to Δ-858 ~ 862 and Δ-946 ~ 950 from the intact promoter and transforming them into *Arabidopsis mir159ab* (Figure 5A), and the T1 transgenic plants also showed a rescued phenotype (Figure 5B). To further characterize the phenotype in more detail, plants were grown in a growth chamber under long-day conditions at 22°C for 3 weeks, the phenotype showed that there was no distinct difference compared to transgenic plants with a promoter of 1.5 kb. Overall, these results suggest that the MybSt1 *cis*-element of the *miR159* promoter was not the key element in rescuing the *mir159ab* phenotype.

However, we found that although the expression level of miR159a.1–3 was increased compared to that of *mir159ab* in MybSt1-mutated transgenic plants, which rescued the phenotype, the changed level was less than 1.5 kb in promoter transgenic lines (Figure 5C). These results indicated that MybSt1 *cis*-elements are responsible for promoter activity but are not the main factors. Furthermore, consistent with the transcript translational repression mechanism, all the MybSt1-motif transgenic plants with fully rescued phenotypes had slightly higher levels of *MYB33* in MybSt1-mutated lines than in the 1.5 kb promoter transgenic lines, although *MYB33* was suppressed in MybSt1-motif transgenic plants compared to those with the *mir159ab* phenotype (Figure 5D).

## Discussion

In plants, many miRNA-target relationships are ancient, and they appear to play fundamental roles in plant growth and development. Despite extensive analyses, there has been little investigation into the properties of structural determinants that govern their efficacy. Using the miR159-MYB33/MYB65 module as a model system in this case, we examined the new nested miR159 structure properties that control the efficacy of silencing by a highly conserved plant miRNA.

### Sequence alignment and phylogenetic analysis reveal that the nested miR159 structure is broadly conserved across land plants

From an evolutionary point of view, miRNAs are usually divided into highly conserved ancient miRNAs and weakly conserved species-or clade-specific miRNAs (Voinnet, 2009). The phylogeny of highly conserved miRNA genes, such as the ancient *MIR159* gene family, which plays an important role in plant growth, is largely unknown (Palatnik et al., 2007). According to previous studies, the precursors of miR159a in *Arabidopsis* had two additionally highly conserved regions outside of the miRNA/miRNA* duplex base-paired region (Palatnik et al., 2003), while cloning and northern blot analysis confirmed the existence of a second small RNA in the precursor of miR159.2 from *P. voulgaris*, and studies in *Arabidopsis* led us to functionally characterize its silencing efficacy (Arenas Huertero et al., 2009; Zhang et al., 2010). *MIR159a* homologs identified in this study were compared, and two blocks of sequence conservation were observed. Comparison of different miR159a precursor sequences revealed an extended occurrence of miR159a.2 (Li et al., 2011). The overall sequence of miR159a.2–5/miR159a.2–3 itself has been conserved during plant diversification with a limited number of changes that still allow comparison of the small RNA sequences (Figure 3; Axtell and Bartel, 2005; Li et al., 2011; Chorostecki et al., 2017).

To further explore the evolutionary relationships of miR159a, we aligned a large number of land plant MIR159 stem-loops and constructed a phylogenetic tree using maximum likelihood (ML). We observed two groups of *MIR159a* in our consensus tree. Group I included dicots, while the monocots formed Group II. Similarly, alternative conserved region (ACR) miRNAs also have two main types (branched and long stems, Supplementary Figure S3), indicating different selection pressures imposed on monocot and dicot miRNAs on the same stem-loop. Our results support a common origin of *MIR159a* aspects because another duplex outside miR159 is highly conserved in most *MIR159* stem-loops across land plants (Chorostecki et al., 2017). Furthermore, the partitions indicated by the conserved areas are compatible with the phasing of mature MIR159 miRNAs from mosses to flowering plants, which has been seen in previous research (Figure 1; Addo Quaye et al., 2009; Bologna et al., 2009). These findings show that MIR159 was derived from a common phased stem-loop RNA, similar to those recently found in the green alga *Chlamydomonas reinhardtii* and rice (Zhao et al., 2007; Zhu et al., 2008).

MiRNAs are important in many aspects of plant growth (Rubio Somoza et al., 2009). As in the shared ancestor of all embryophytes, miR159 was discovered to be one of the eight closely conserved miRNA families (Cuperus et al., 2011). Transgenic plants constitutively overexpressing miR159 have been constructed in *Arabidopsis*, rice, wheat, and *Gloxinia*. In all these species, our miR159a resulted in similar phenotypes, including in the 3*´-*UTR-overexpressing *Arabidopsis* plants, which had a larger leaf size and delayed flowering, suggesting an evolutionarily conserved function of miR159 and *MYB* target genes in plant development. miR159a-3*´-*UTR-overexpressing *Arabidopsis* have effect on phenotype and showed a decreased level of *MYB33* mRNA, which is consistent with previous reports (Achard et al., 2004). Similarly, in rice, overexpression of miR159 results in delayed head formation mainly due to decreases in *OsGAMYB* and *OsGAMYBL1* (Tsuji et al., 2006). In contrast, another piece of evidence showed that overexpressed miR159 downregulated *MYB101* but not *MYB33* and *MYB65* (Schwab et al., 2005). Based on these findings, the biological function of miR159 in plant growth and development appears to be determined by a complex mechanism.

### 5′-UTR roles in the determination of the specific promoter length regulating miR159a

It is well known that the 5*′-*untranslated region (5*′*-UTR) plays a key role in transcriptional and posttranscriptional regulation of gene expression (Hua et al., 2001; Hulzink et al., 2002). Plant pri-miRNA transcription is identical to that of protein-coding genes, the majority of which are transcribed from their own transcriptional units termed *MIR* genes, whose genome sequences are usually located at intergenic regions of protein-coding genes and have their own promoters and independent regulatory patterns (Griffiths Jones et al., 2008; Meyers et al., 2008; Nozawa et al., 2012). In addition, short open reading frame sequences involved in the synthesis of regulatory peptides have recently been discovered in pri-miRNAs and can facilitate the aggregation of their own mature miRNAs (Lauressergues et al., 2015).

In our study, the ath-miR159a promoter from *A. thaliana* was isolated and characterized by deletion analysis in *mir159ab* transgenic plants. Following deletion analysis, we found that constructs with the ath-miR159a promoter (−3.0 kb) and its 5′ deletion fragments (−3.0 to −1.0 kb) could rescue the phenotype of *mir159ab* and deregulate the *MYB33/65* gene. In contrast, the truncated −0.7 kb promoter showed slightly higher expression of *MYB33* than the −1.0 kb promoter and partially rescued the phenotype, indicating that important regulating elements located between −0.7 and −1.0 kb govern the expression of miR159. However, all the truncated constructs longer than 1.0 kb fully rescued the *mir159ab* phenotype, which suggested that the 1.0 kb core functional segment of the miR159a promoter was a potential regulator of *MIR159a* (Figure 5).

**Figure 6:**
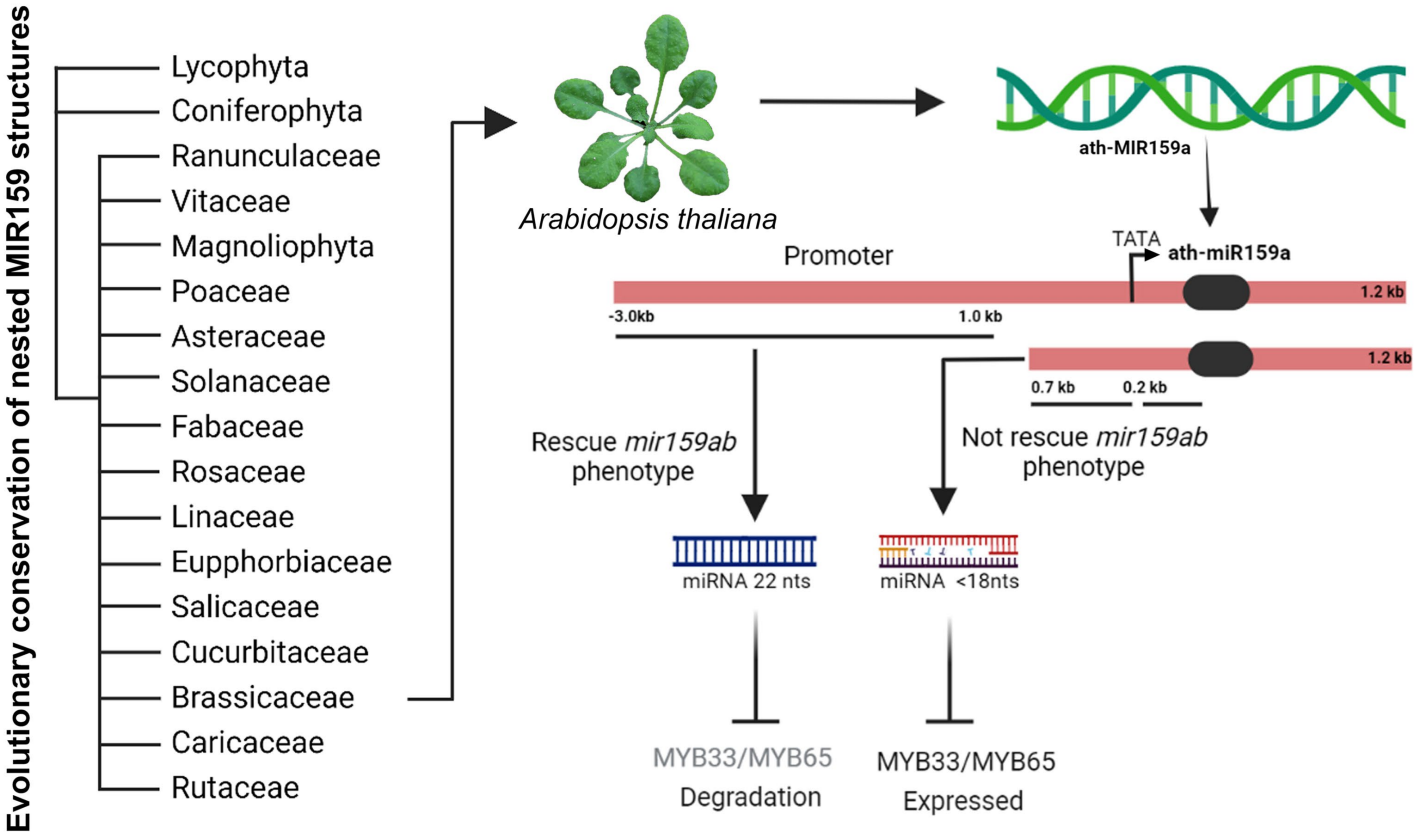
Model figure representing the evolutionary conservation of nested MIR159 structures in plants and functional characterization of ath-miR159a promoter in Arabidopsis.

Promoters usually regulate the intensity of gene expression through the interaction of some specific *cis*-acting elements on the sequence with their interacting proteins, such as transcription factors. Our bioinformatic analysis showed that the 3.0 kb sequence, including the 1.0 and 0.7 kb regions, contains various elements, including two MybSt1 motifs (Figure 5A). Members of the MYB-related protein family are known to be DNA binding proteins, and the binding site for MybSt1 contains the core element GGATA (Baranowskij et al., 1994). Interestingly, we found two MybSt1 binding sites at positions −858 and −946. Thus, we speculate that MYB could bind to these sequences, enhance the activation of promoters, and form a negative feedback loop by suppressing miR159a. It has been confirmed previously that MybSt1 binding sites in the promoter are important and contribute to its promoter activity (Baranowskij et al., 1994). Based on the above results, we speculated that the MybSt1 elements may be important factors contributing to the high promoter activity of 1.0 kb and enhanced activities of the 858 and 946-bp fragments. Moreover, the deletion of the −858, −946, and both (−858 ~ −946) sequences resulted in a decrease in miR159a.1–3, thus causing little change by increasing the *MYB33* expression activity of *Arabidopsis* transgenic plants, although the phenotype was almost rescued. To conclude, the findings reported here have identified specific regions in the 5′UTR required for regulating the expression of miR159a and presented in model (Figure 6). These MybSt1 *cis*-acting elements have affected the expression of miR159a; therefore, it is interesting to determine which *MYB* transcription factor will bind to this site to elevate miR159. Overall, our results revealed the conserved structure of miRNA159 among different species and rendered the regulation of the nested structure of each distinct miR159.

## Conclusion

In summary, our results from sequence alignment and phylogenetic analysis suggest that the nested miR159 structure is broadly conserved in their stem-loops across land plants. Further, genetic analysis proposed that a 1.0 kb promoter can completely rescue the *mir159ab* phenotype and miR159.2 accumulation indicates that this may have regulatory functions that need to be tested.

## Data availability statement

The original contributions presented in the study are included in the article/Supplementary material, further inquiries can be directed to the corresponding author.

## Author contributions

ZT designed the experiments and managed the project. MI, TL, ZW, MW, SL, XG, AW, and SFL performed gene cloning and functional analysis. TL performed the data analyses. MI, MZ, and ZT wrote the manuscript. All authors contributed to the article and approved the submitted version.

## Funding

This work was supported by the National Key Research and Development Program of China (2021YFD1201101), the National Natural Science Foundation of China (32172053, 32001501).

## Conflict of interest

The authors declare that the research was conducted in the absence of any commercial or financial relationships that could be construed as a potential conflict of interest.

## Publisher’s note

All claims expressed in this article are solely those of the authors and do not necessarily represent those of their affiliated organizations, or those of the publisher, the editors and the reviewers. Any product that may be evaluated in this article, or claim that may be made by its manufacturer, is not guaranteed or endorsed by the publisher.

## Footnotes

^1^ https://rsb.info.nih.gov/ij/

^2^ https://www.dna.affrc.go.jp/PLACE/?action=newplace

^3^ http://www.mirbase.org

^4^ http://www.arabidopsis.org/
