# Supplementary material for: Evolutionary conservation of nested MIR159 structural microRNA genes and their promoter characterization in Arabidopsis thaliana
Source: Front Plant Sci. 2022 Jul 26;13:948751. doi: 10.3389/fpls.2022.948751 (PMC9361848; doi:10.3389/fpls.2022.948751)
Supplement: Supplementary file 2 [file Presentation_1.PPTX]

## Slide 1
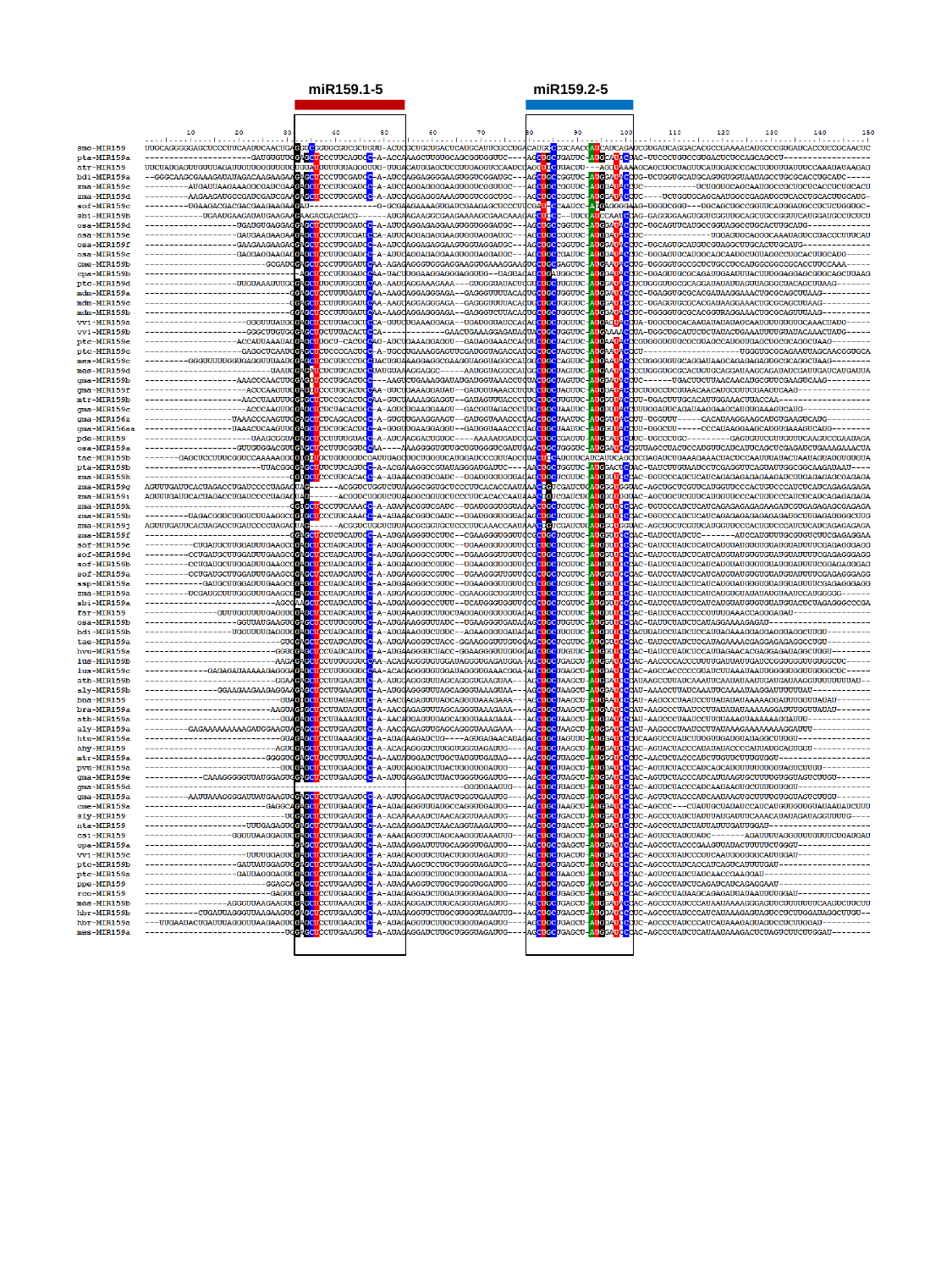

miR159.1-5
miR159.2-5

## Slide 2
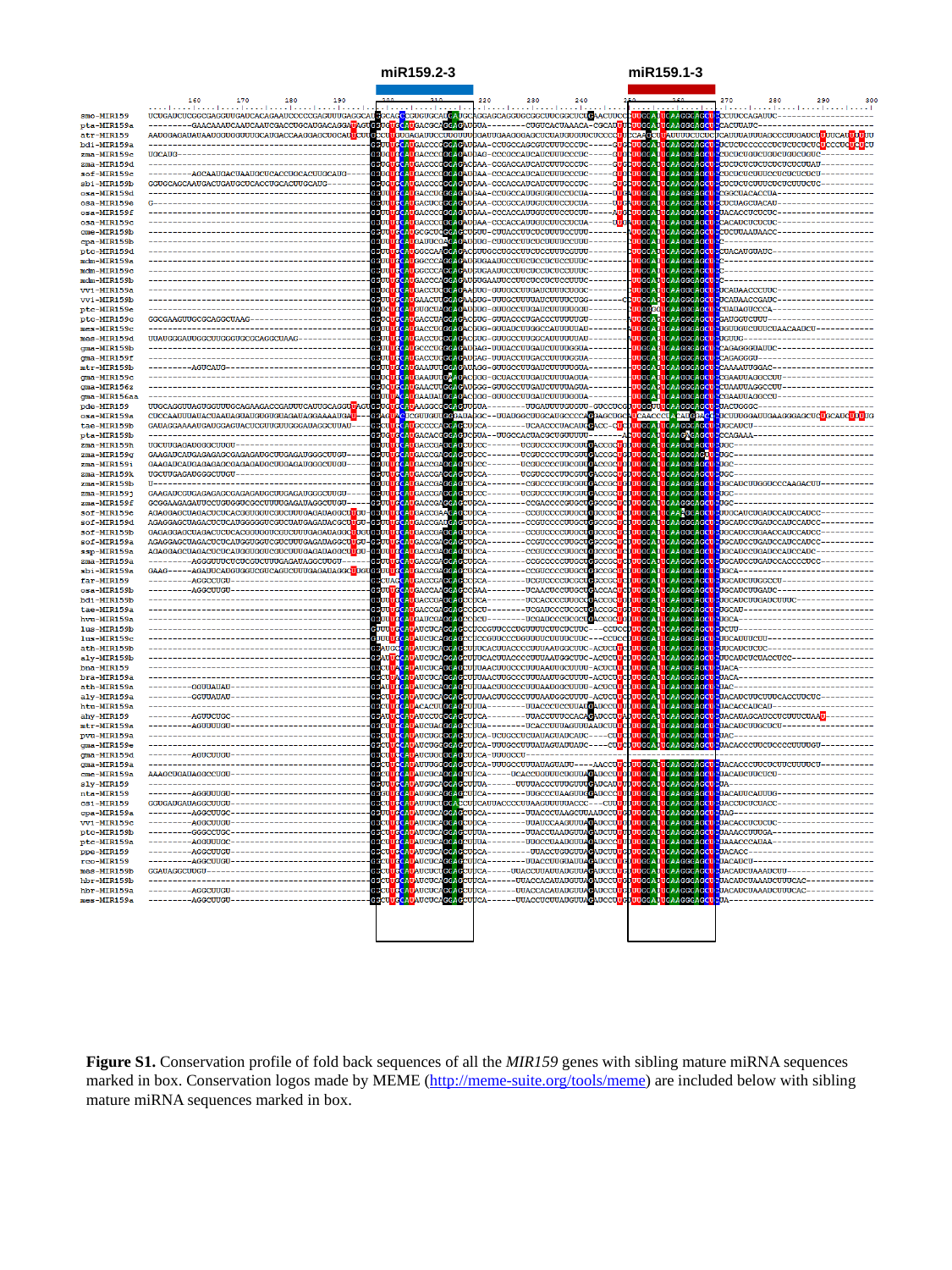

miR159.2-3
miR159.1-3
Figure S1. Conservation profile of fold back sequences of all the MIR159 genes with sibling mature miRNA sequences marked in box. Conservation logos made by MEME (http://meme-suite.org/tools/meme) are included below with sibling mature miRNA sequences marked in box.
