# Supplementary material for: Evolutionary conservation of nested MIR159 structural microRNA genes and their promoter characterization in Arabidopsis thaliana
Source: Front Plant Sci. 2022 Jul 26;13:948751. doi: 10.3389/fpls.2022.948751 (PMC9361848; doi:10.3389/fpls.2022.948751)
Supplement: Supplementary file 3 [file Presentation_2.pptx]

## Slide 1
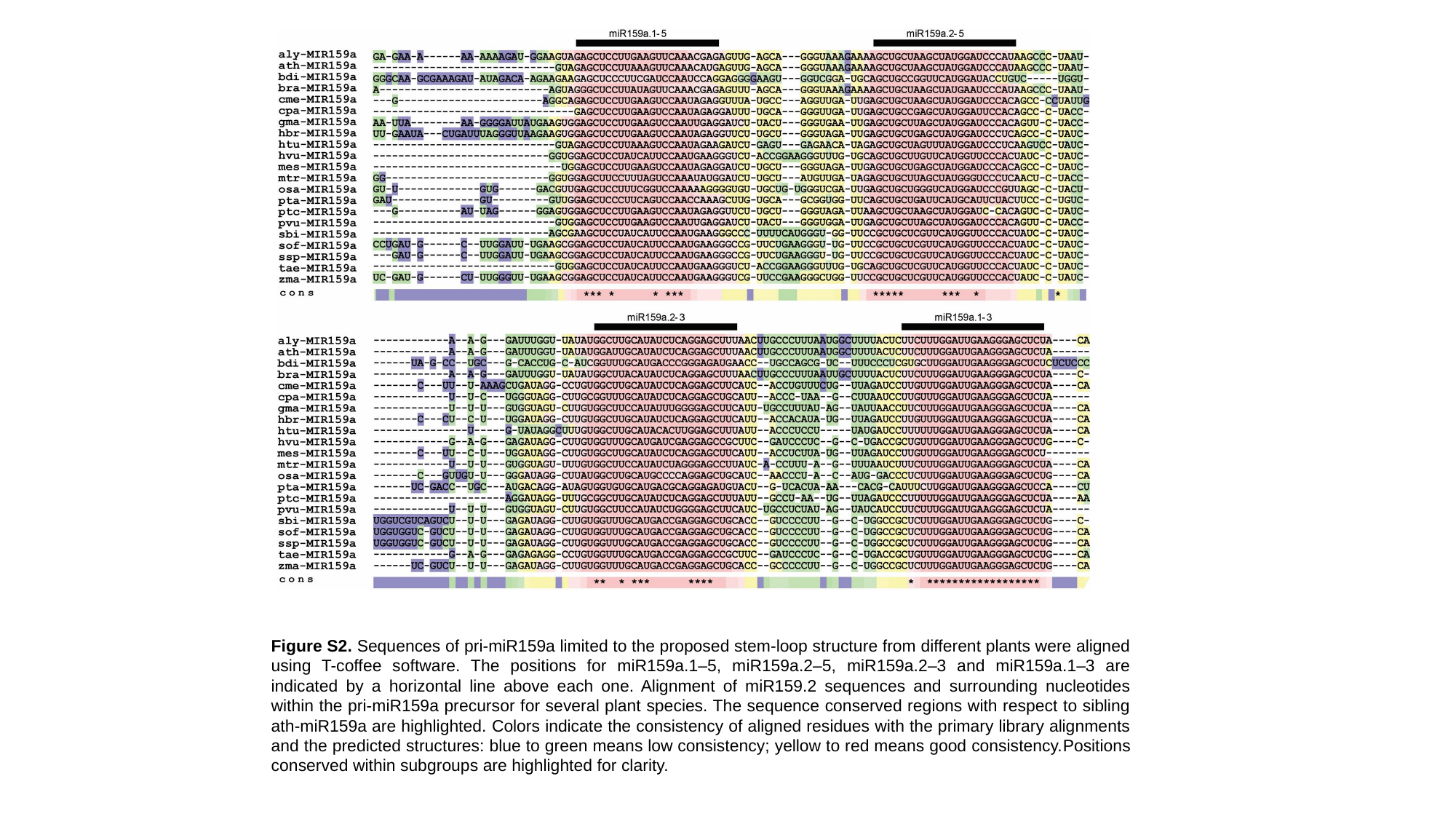

Figure S2. Sequences of pri-miR159a limited to the proposed stem-loop structure from different plants were aligned using T-coffee software. The positions for miR159a.1–5, miR159a.2–5, miR159a.2–3 and miR159a.1–3 are indicated by a horizontal line above each one. Alignment of miR159.2 sequences and surrounding nucleotides within the pri-miR159a precursor for several plant species. The sequence conserved regions with respect to sibling ath-miR159a are highlighted. Colors indicate the consistency of aligned residues with the primary library alignments and the predicted structures: blue to green means low consistency; yellow to red means good consistency.Positions conserved within subgroups are highlighted for clarity.

## Slide 2
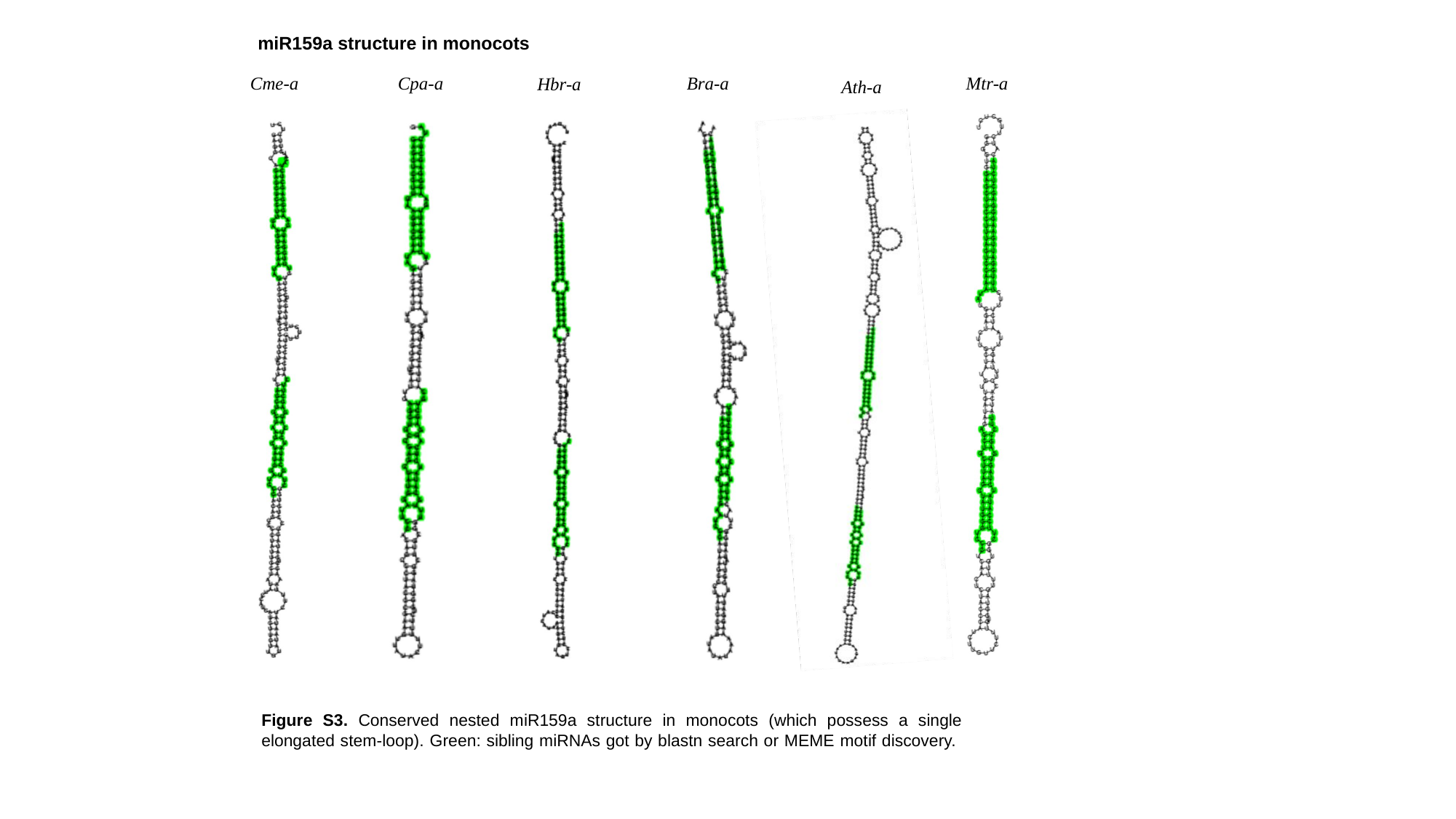

miR159a structure in monocots
Bra-a
Mtr-a
Cpa-a
Cme-a
Hbr-a
Ath-a
Figure S3. Conserved nested miR159a structure in monocots (which possess a single elongated stem-loop). Green: sibling miRNAs got by blastn search or MEME motif discovery.

## Slide 3
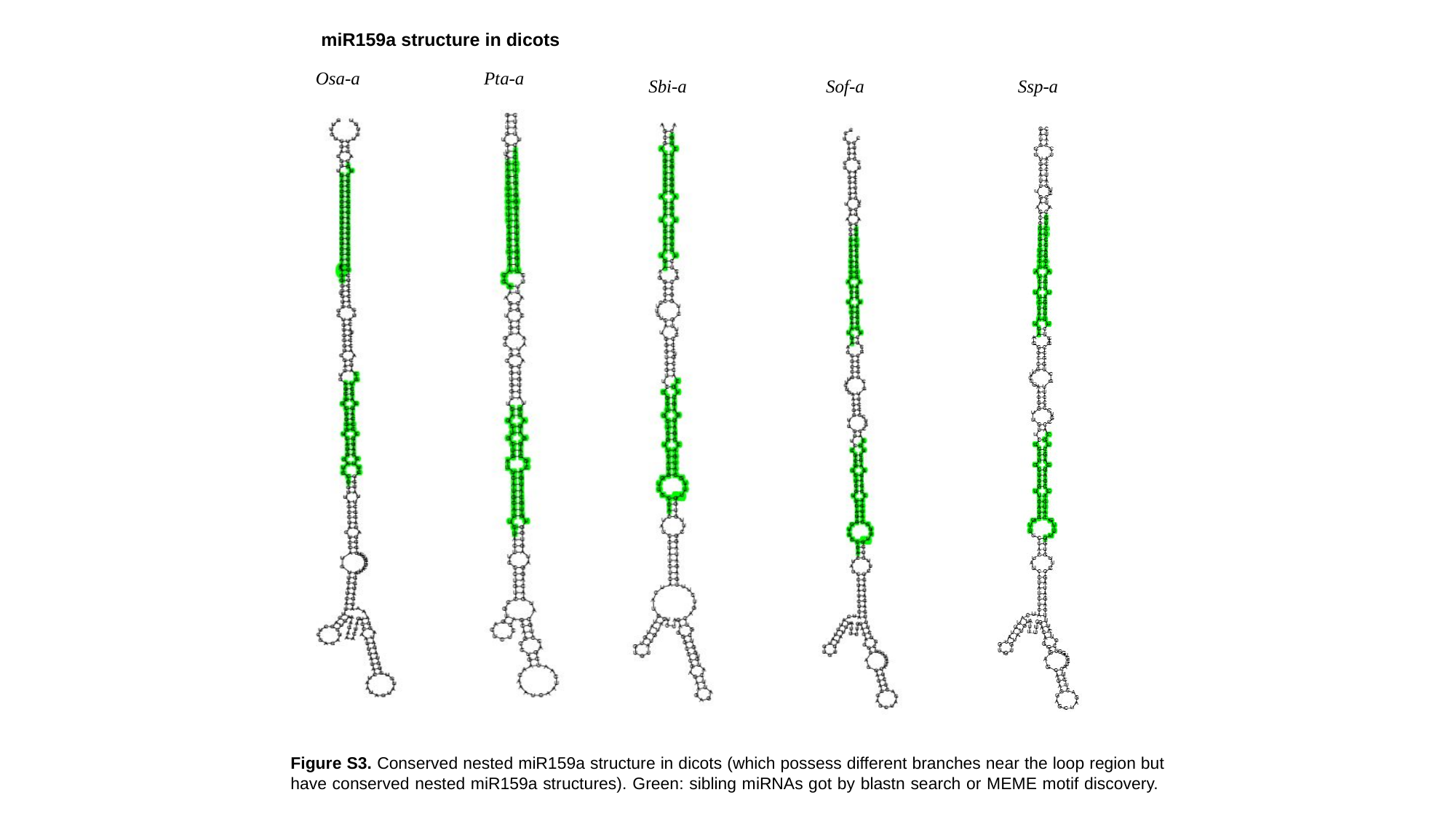

miR159a structure in dicots
Osa-a
Pta-a
Ssp-a
Sbi-a
Sof-a
Figure S3. Conserved nested miR159a structure in dicots (which possess different branches near the loop region but have conserved nested miR159a structures). Green: sibling miRNAs got by blastn search or MEME motif discovery.
